# Generalizable EHR-R-REDCap pipeline for a national multi-institutional rare tumor patient registry

**DOI:** 10.1093/jamiaopen/ooab118

**Published:** 2022-01-07

**Authors:** Sophia Z Shalhout, Farees Saqlain, Kayla Wright, Oladayo Akinyemi, David M Miller

**Affiliations:** 1 Division of Hematology/Oncology, Massachusetts General Hospital, Boston, Massachusetts, USA; 2 Harvard Medical School, Boston, Massachusetts, USA; 3 Department of Dermatology, Massachusetts General Hospital, Boston, Massachusetts, USA

**Keywords:** EHR, rare tumor registry, Merkel Cell Carcinoma, REDCap, R statistical software, patient registries

## Abstract

**Objective:**

To develop a clinical informatics pipeline designed to capture large-scale structured Electronic Health Record (EHR) data for a national patient registry.

**Materials and Methods:**

The EHR-R-REDCap pipeline is implemented using R statistical software to remap and import structured EHR data into the Research Electronic Data Capture (REDCap)-based multi-institutional Merkel Cell Carcinoma (MCC) Patient Registry using an adaptable data dictionary.

**Results:**

Clinical laboratory data were extracted from EPIC Clarity across several participating institutions. Laboratory values (Labs) were transformed, remapped, and imported into the MCC registry using the EHR labs abstraction (eLAB) pipeline. Forty-nine clinical tests encompassing 482 450 results were imported into the registry for 1109 enrolled MCC patients. Data-quality assessment revealed highly accurate, valid labs. Univariate modeling was performed for labs at baseline on overall survival (*N* = 176) using this clinical informatics pipeline.

**Conclusion:**

We demonstrate feasibility of the facile eLAB workflow. EHR data are successfully transformed and bulk-loaded/imported into a REDCap-based national registry to execute real-world data analysis and interoperability.

## Lay Summary

Healthcare data collected during routine clinical care in patient electronic health records (EHRs) can be curated and organized into disease-focused registries. For example, the Merkel Cell Carcinoma Patient Registry is a multi-institutional national database that utilizes real-world health care data to inform best practices, improve patient outcomes, and test hypotheses that require large data samples. However, many challenges arise when capturing patient-level details/data from the health records especially when this data must be aggregated across multiple institutions. Here, we describe a pipeline that (1)pulls large-scale data from the EHRs, (2)wrangles and remaps the data in a format appropriate for the database using R statistical software, and (3)imports the data in bulk for thousands of patients at a time into Research Electronic Data Capture (REDCap), the electronic data capture system that houses the data. The EHR-R-REDCap pipeline can be utilized to capture many patient data features from the participating institutions. Here, we demonstrate eLAB, the EHR-R-REDCap pipeline utilized by our Merkel Cell Carcinoma Patient Registry to capture thousands of laboratory values from the EHR for thousands of patients. We provide source code, examples, and instructions for adaptability for other clinical research projects that aim to collect large-scale laboratory data from the EHR.

## INTRODUCTION

**Figure 1.**
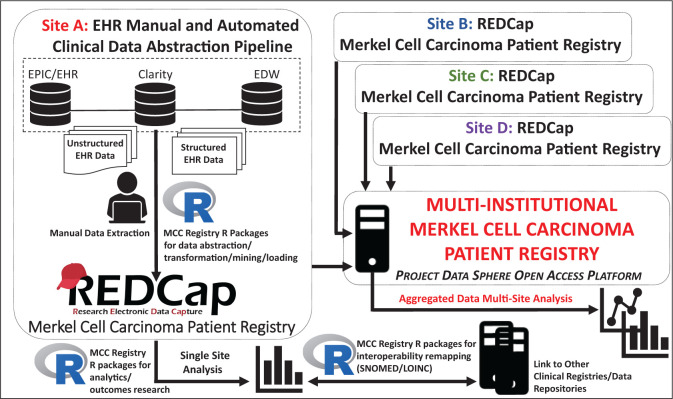
Schematic overview of the multi-institutional MCCPR. The EHR-R-REDCap pipeline is implemented at each site (eg Site A–D) to allow for rapid remapping and transformation of structured data for import into REDCap from a variety of sources using several MCCPR-driven R statistical software scripts and registry-driven R packages. This pipeline augments the manual data abstraction implemented for the capture of nonstructured EHR data. Data collection and single-site analysis are streamlined at each institution. The multisite aggregated data will be hosted on Project Data Sphere’s (PDS) open source platform.

Patient registries focus on the collection of clinically relevant data around a target population. In the era of precision medicine, national patient registries have gained momentum.^1–3^ Furthermore, there exists a critical need to develop rare tumor patient registries to better characterize the natural history of these cancers.^4–6^ The national Merkel Cell Carcinoma (MCC) Patient Registry (MCCPR) was developed to record outcomes and events in patients diagnosed with this rare and aggressive skin cancer.^7^^,^^8^ This will enable multiple investigators to examine real-world data to improve patient outcomes and identify best practices. The development of the MCCPR is an evolving effort led by researchers and clinical investigators from academia, industry, and regulatory science. Data are captured at participating sites using registry-specific instruments developed in the Research Electronic Data Capture (REDCap) system, a widely used web-based platform.^9^^,^^10^ The goal is to disseminate the multi-institutional aggregated data on the first publicly held national MCCPR, utilizing Project Data Sphere’s open-access platform^7^ (Figure  1).

Registry data collection is a time-intensive process requiring electronic health record (EHR) navigation and manual chart review. Automated abstraction of structured EHR data allows for the rapid generation of large-scale data to drive registry research. We adopted accessible, affordable software, REDCap and R, to lower technical and economic barriers for participating sites^9–11^ to remap and load/import data into the REDCap-based MCCPR using the data dictionary (DD). The pipeline facilitates large-scale registry data collection, analysis, and interoperability/standardization for many features including demographics, and medications. Importantly, this pipeline anticipates and allows for modifications of the scripts/packages as site-specific needs arise to accommodate variability in the sources/format of initial EHR data input.

The objective of this study is to demonstrate and evaluate the EHR-R-REDCap pipeline with the EHR labs abstraction (eLAB) pipeline, developed for importing structured EHR clinical lab data using the registry REDCap DD. We implemented eLAB to extract lab data from the EHR of 1109 registry subjects from several institutions including Massachusetts General Hospital, Dana-Farber Cancer Center, and Brigham and Women’s Hospital and several satellite/community sites. We also performed proof-of-concept exploratory outcomes research on a test cohort (*N* = 176). Finally, we evaluated the capture time and data quality/accuracy across patients from different sites in comparison to manually abstracted data and the EHR.

We developed and provide here: (1) the DD to allow users to adopt the MCCPR REDCap labs instrument, (2) key-value/lookup tables for remapping ∼300 EHR lab-subtypes into the 35 registry-specific laboratory values (labs) of interest, (3) eLAB source code (https://github.com/TheMillerLab/eLAB), and (4) a sample data set for demonstration purposes. Furthermore, we provide (5) a detailed dynamic report as an R-markdown^12^ file annotating all functions/R-script. We highlight examples of upfront script modifications that may be needed to accommodate bulk lab data pulls from other EHR data warehouse (EDW) sites/sources. Downstream script is largely preserved when utilizing the accompanying DD. Importantly, these resources may be adopted by other clinical research projects/registries to minimize developer efforts.

## MATERIALS and METHODS

### eLAB development and source code (R statistical software)

eLAB is written in R^11^ (version 4.0.3) and utilizes the following packages for processing: DescTools, REDCapR, reshape2, splitstackshape, readxl, survival, survminer, and tidyverse. Source code for eLAB can be downloaded directly (https://github.com/TheMillerLab/eLAB).

eLAB reformats EHR data abstracted for an identified population of patients (eg medical record numbers (MRNs)/name list) under an Institutional Review Board (IRB)-approved protocol. The MCCPR does not host MRNs/names and eLAB converts these to MCCPR-assigned record identification numbers (record_id) before import for de-identification.

Functions were written to remap EHR bulk lab data pulls/queries from several sources including Clarity/Crystal reports or institutional EDW including Research Patient Data Registry (RPDR) at Mass General Brigham (MGB). The input, a csv/delimited file of labs for user-defined patients, may vary. Thus, users may need to adapt the initial data wrangling script based on the data input format. However, the downstream transformation, code-lab lookup tables, outcomes analysis, and Logical observation identifiers names and codes (LOINC) remapping are standard for use with the provided REDCap DD. The available R-markdown provides suggestions and instructions on where or when upfront script modifications may be necessary to accommodate input variability.

**Figure 2.**
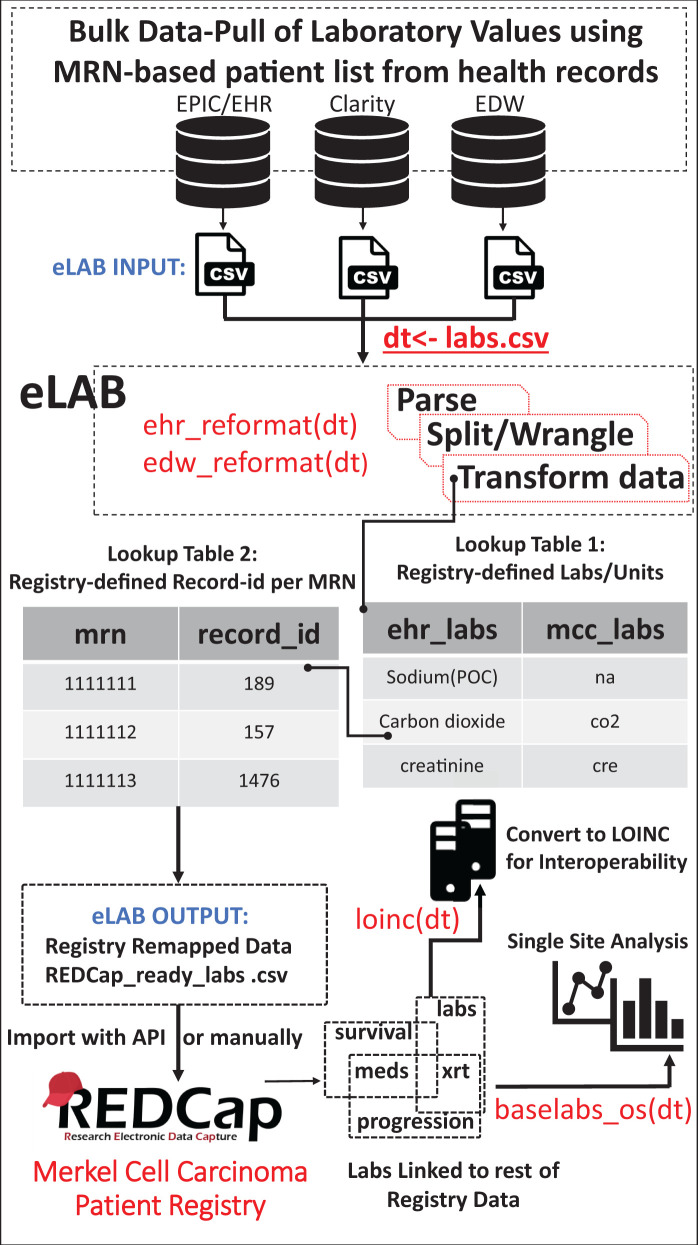
Schematic overview of the eLAB clinical informatics pipeline. eLAB is designed to take as input csv data from several EHR/EDW sources and may be adapted for other site-specific inputs. Once delimited data are assigned as R object “dt,” a single-line command is used to transform the data into the REDCap-ready registry configuration for import based on source type (eg ehr_reformat(), or edw_reformat()). eLAB is designed to de-identify the patient names/medical record numbers (MRNs) with registry-specific record identification (record_id) numbers. Furthermore, 300 subtypes of laboratory tests (ehr_labs) are remapped into 35 registry fields (mcc_labs). Once imported into the registry via an output csv file or using REDCap API token, single-line commands are used for outcomes research and analysis (baselabs_os()), as well as remapping for interoperability with standardized SNOMED or LOINC code (loinc()). LOINC remapping is an optional feature provided by eLAB to allow the MCCPR data to be linked to other non-MCCPR clinical research efforts/registries that may utilize LOINC. Using a lookup table dependent on the MCCPR data dictionary, eLAB remaps 1 LOINC code per 1 data dictionary field variable name only after the data has successfully been cleaned, remapped, transformed, and imported.

The eLAB pipeline takes several inputs. For example, the input for use with the “ehr_format(dt)” single-line command is nontabular data assigned as R object “dt” with 4 columns: (1) Patient Name (MRN), (2) Collection Date, (3) Collection Time, and (4) Lab Results wherein several lab panels are in one data frame cell. A mock data set in this “untidy-format” is provided for demonstration purposes (https://github.com/TheMillerLab/eLAB) (Figure  2, Supplemental Materials).

Bulk lab data pulls often result in subtypes of the same lab. For example, potassium labs are reported as “Potassium,” “Potassium-External,” “Potassium(POC),” “Potassium, whole-bld,” “Potassium-Level-External,” “Potassium, venous,” and “Potassium-whole-bld/plasma.” eLAB utilizes a key-value lookup table with ∼300 lab subtypes for remapping labs to the DD code. eLAB reformats/accepts only those lab units predefined by the registry DD. The lab lookup table is provided for direct use or may be re-configured/updated to meet end user specifications. eLAB is designed to remap, transform, and filter/adjust value units of semistructured/structured bulk laboratory values data pulls from the EHR to align with the predefined code of the DD.

### Data dictionary

**Figure 3.**
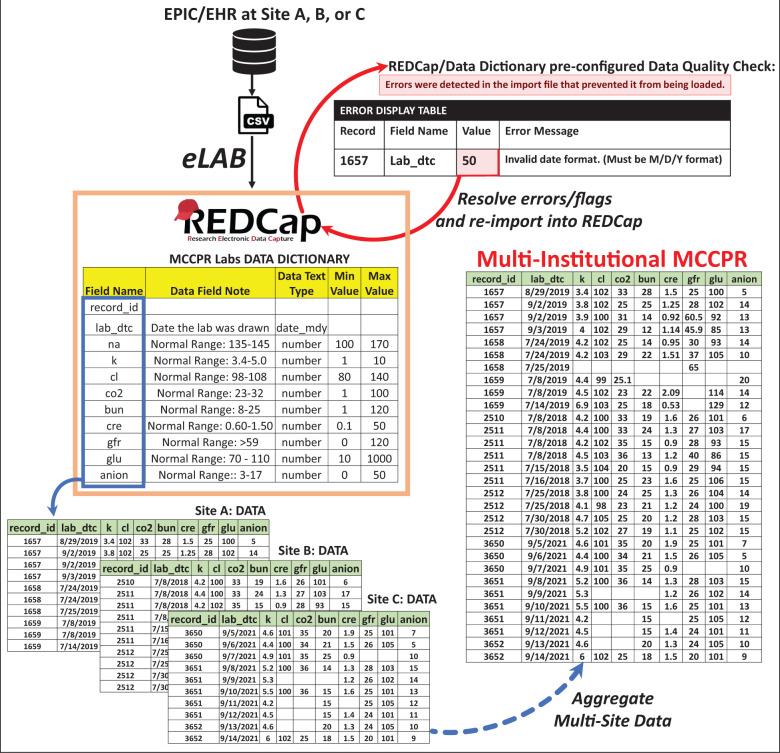
eLAB and the data dictionary to harmonize multi-institutional data aggregation. The data dictionary, once uploaded into REDCap, creates the lab capture system or the “Labs instrument.” eLAB is designed to reformat and normalize laboratory values and units that are bulk-pulled from the EHR. eLAB transforms the data into a format predefined by the data dictionary and its associated variable codes. eLAB performs the primary data cleansing steps. However, one final quality check is also utilized at the very end during import of data. If an attempt is made to import any data that is incorrectly reformatted (ie numerical value for a date field that is not in the acceptable M/D/Y format), an error is set off during the final stage of import by the REDCap scaffold that is associated with the preconfigured design of the data dictionary. Data with errors will fail to import into REDCap. The REDCap data import tool will display the data that does not conform to the configuration designated by the data dictionary, and it will be flagged, alongside error messages that provide guidance on how to resolve the issue. The data will have to be re-evaluated and corrected. Only when the data are free of errors and all issues are resolved, a successful upload/import into REDCap can occur. With every site in the multi-institutional registry utilizing eLAB and the exact same data dictionary, aggregation of the data is straightforward and only requires combining/appending the outputs of each site together (ie each site will combine individual “.csv file” into one large multisite “.csv file” for the final aggregated data). Multisite data aggregation is facilitated by each participating site (1) utilizing eLAB for transformation and normalization with (2) the accompanying data dictionary.

EHR clinical laboratory data are captured in REDCap using the “Labs” repeating instrument (Supplemental Figures 1 and 2). The DD is provided for use by researchers at REDCap-participating institutions and is optimized to accommodate the same lab type captured more than once on the same day for the same patient. The instrument captures 35 clinical lab types (Table  1). The DD serves several major purposes in the eLAB pipeline. First, it defines every lab type of interest and associated lab unit of interest with a set field/variable name. It also restricts/defines the type of data allowed for entry for each data field, such as a string or numerics. The DD is uploaded into REDCap by every participating site/collaborator and ensures each site collects and codes the data the same way. Automation pipelines, such as eLAB, are designed to remap/clean and reformat data/units utilizing key-value lookup tables that filter and select only the labs/units of interest. eLAB ensures the data pulled from the EHR contains the correct unit and format preconfigured by the DD. The use of the same DD at every participating site ensures that the data field code, format, and relationships in the database are uniform across each site to allow for the simple aggregation of the multisite data. For example, since every site in the MCCPR uses the same DD, aggregation is efficient and different site csv files are simply combined (Figure  3).

**Table 1.** Proof-of-concept univariable model (*N* = 176, Hazard Ratios with a *P* value < 0.05 shown in bold)

	Overall survival	
Laboratory tests	Hazard ratio (95% CI)	*P* value
Electrolytes/renal/glucose		
Sodium (na)	0.9332 (0.82–1.06)	.300
Potassium (k)	1.7750 (0.72–4.40)	.215
Chloride (cl)	0.9146 (0.82–1.02)	.097
Carbon dioxide (co2)	0.9600 (0.84–1.1)	.560
Blood urea nitrogen (bun)	**1.0444 (1.02–1.07)**	**.000182**
Creatinine (cre)	**1.8452 (1.34–2.55)**	**.000202**
Estimated glomerular filtration rate (gfr)	**0.9656 (0.95–0.98)**	**.000195**
Blood glucose (glu)	1.0030 (0.99–1.01)	.575
Anion gap (anion)	1.112 (0.96–1.29)	.149
General chemistries		
Albumin (alb)	0.7204 (0.18–2.92)	.646
Total bilirubin (tbili)	0.1340 (0.01–1.90)	.137
Calcium (ca)	0.8072 (0.38–1.70)	.574
Total protein (tp)	0.6961 (0.20–2.38)	.563
Liver function tests		
Alanine aminotransferase (sgpt)	0.9798 (0.92–1.05)	.535
Aspartate aminotransferase (sgot)	1.0078 (0.93–1.08)	.827
Alkaline phosphatase (alkp)	1.0116 (0.99–1.03)	.122
Globulin (glob)	1.0763 (0.35–3.29)	.897
Hematological studies		
White blood cells (wbc)	0.9891 (0.94–1.04)	.661
Red blood cells (rbc)	0.5838 (0.26–1.32)	.197
Hemoglobin (hgb)	0.9562 (0.74–1.23)	.726
Hematocrit (hct)	0.9897 (0.90–1.10)	.836
Mean corpuscular volume (mcv)	**1.0994 (1.02–1.19)**	**.0137**
Mean corpuscular hemoglobin (mch)	1.1878 (0.95–1.48)	.129
Mean corpuscular hemoglobin conc. (mchc)	0.9195 (0.70–1.21)	.551
Platelet count (plt)	0.9949 (0.99–1.00)	.127
Mean platelet volume (mpv)	1.1619 (0.82–1.65)	.405
Red cell distribution width (rdw)	1.1377 (0.89–1.46)	.312
Percent neutrophils (%) (neut)	0.9926 (0.96–1.02)	.615
Absolute neutrophil count (anc)	0.8880 (0.61–1.29)	.537
Percent lymphocytes (%) (lymp)	1.0884 (0.98–1.04)	.555
Absolute lymphocyte count (alc)	0.9955 (0.95–1.04)	.837
Percent monocytes (%) (mon)	**1.1476 (1.01–1.31)**	**.041**
Absolute monocyte count (amc)	**3.9374 (1.22–12.73)**	**.022**
Percent eosinophils (%) (eosp)	0.8099 (0.58–1.13)	.217
Absolute eosinophil Count (aec)	0.0468 (0.0003–6.46)	.223
Percent basophils (%) (basop)	0.3741 (0.08–1.77)	.214
Absolute basophil count (abc)	0.00012 (2e-12–7297)	.324
Percent granulocytes, immature (immgranp)	0.4491 (0.03–6.28)	.552
Absolute immature granulocytes Count (agc)	0.0005 (4e-16–6.7e8)	.593
Percent nucleated RBCs (nrbc)	3.8e-16 (0–∞)	.997
Neutrophil to lymphocyte ratio (nlr)	1.034 (0.78–1.37)	.815
Lactate dehydrogenase (ldh)	1.002 (0.99–1.02)	.707

### Study cohort

This study was approved by the MGB IRB Protocol# 2019P002459. Search of the EHR was performed to identify patients diagnosed with MCC between 1975 and 2021 (*N* = 1109) for inclusion in the MCCPR. Subjects diagnosed with primary cutaneous MCC between 2016 and 2019 (*N* = 176) were included in the test cohort for exploratory studies of lab result associations with overall survival (OS) using eLAB.

### Statistical analysis

OS is defined as the time from date of MCC diagnosis to date of death. Data were censored at the date of the last follow-up visit if no death event occurred. Univariable Cox proportional hazard modeling was performed among all lab predictors. Due to the hypothesis-generating nature of the work, *P* values were exploratory, and Bonferroni corrections were not applied.

## RESULTS

Clinical labs for 1109 registry subjects were extracted from EPIC Clarity and produced >500 000 lab values. eLAB was utilized to remap results using the REDCap DD, de-identify the patients with a predefined MCCPR “record_id,” and remove nonimportable characters such as “refused,” and “canceled.” Lab subtypes were remapped under the MCCPR lab categories and transformed into a REDCap-ready importable configuration. Precisely, 482 450 lab values for 1109 patients were successfully imported.

We assessed the accuracy of the eLAB pipeline by evaluating the imported results compared to those manually collected by two registry-trained data abstractors. REDCap start/stop date-timestamp fields were implemented to measure time directly spent on manually abstracting structured lab data, including EHR navigation time. Abstractors spent a total of 1458.4 minutes collecting 8043 lab values on 30 patients across the different sites. On average, 5.5 laboratory values per minute were abstracted from EHR. In comparison, ∼3x as many values for the same 30 patients were transformed, remapped, and imported using eLAB in 1.2 minutes. A 97.6% agreement rate was achieved when the two data sets were analyzed for nonagreement, with manual number/typo/date errors accounting for the differences as manually confirmed in the EHR to determine the data set with the error.

Large-scale registry data aggregation is useful for the identification of predictive/prognostic biomarkers, and to uncover patient disease susceptibility. Therefore, proof-of-concept exploratory univariate modeling was performed with eLAB for each lab with date of diagnosis set to baseline for a single-site test cohort (*N* = 176) (Table  1). eLAB calls the appropriate registry survival data from the “subject-status” instrument, date of diagnosis in the “patient characteristics instrument,” and date cutoffs for laboratory data to perform Cox proportional hazards modeling. Blood urea nitrogen (BUN), creatinine, estimated glomerular filtration rate (eGFR), mean corpuscular volume (MCV), as well as percent/absolute monocyte count were found associated with OS in patients with MCC in the test cohort (*P* values < .05 shown in bold, Table  1).

## DISCUSSION

Multi-institutional patient registries are critical in optimizing clinical management and reducing the bias inherent in single-institutional studies. A major barrier for developing national and international programs is the rapid, consistent collection of large-scale data allowing data aggregation and analysis. Here, we develop and evaluate the MCCPR EHR-R-REDCap pipeline using eLAB across several participating sites. In addition, the successful remapping, transformation, import, and analysis of clinical laboratory data on 176 patients was carried out in a fraction of the time required by the standard manual entry pipeline. The time required for manual data capture for 30 patients alone was >1000× fold longer. There are caveats to this time-to-capture comparison. For example, while manual abstraction compared directly to our final automated product is much slower, code development and refinement is a lengthy process and not accounted for in the time-to-capture comparison. Our registry key-value/lookup tables are rarely updated, but if they are often redesigned and re-curated during one’s clinical research efforts, it may increase automated time-to-capture. Furthermore, much of the data capture time required by the EHR-R-REDCap pipeline is dedicated to the importation of the data into REDCap and depends heavily on internet latency. Finally, this system is designed to augment manual abstraction of EHR laboratory data. For example, manual abstractors may instead focus efforts on capturing unstructured lab data from outside hospital (OSH) faxed/scanned reports.

Several strategies have been developed and proposed in the past to automate the retrieval of structured EHR data for import into REDCap repositories including dynamic data pull modules,^13^^,^^14^ and REDCap plugins that connect REDCap directly to the EHR and SFTPs servers.^15^ However, these strategies often require dedicated institutional REDCap personnel to build web services as middleware and require data format setup. Selections are often negotiated with the institution’s REDCap administrators. In addition, each participating site will need to individually remap sourced data to the registry fields. In the EHR-R-REDCap pipeline, the registry defined acceptable source values are provided for participating sites, and users may adjust/add as needed. With eLAB, several EHR values are easily mapped to the same registry code when appropriate and have proven successful across several sites.

Furthermore, proposed methods that rely on REDCap-EHR direct communication fetch data in real-time and need to be adjudicated per value per patient, before importing the data into the project. The EHR-R-REDCap pipeline takes advantage of REDCap’s data import scaffold, allowing for the identification of errors in bulk and user-adjudication before overwriting any previous data stored in the registry. The EHR-R-REDCap pipeline allows for routine bulk pulls during user-defined time windows. For example, in longitudinal studies where data points are routinely updated such as laboratory values, data can be systematically updated once a month only, on all patients simultaneously in the registry. Finally, it may be challenging for each participating institution, including community sites, to map individual institution’s data models to standardized codes. With rare tumors, each site is essential to increase enrollment.

The EHR-R-REDCap pipeline also reduces the barriers for data wrangling and analysis at each site. We demonstrate feasibility of eLAB to carry out exploratory data analysis (Table  1). Conclusions related to MCC are beyond the scope of this work but will be addressed in additional single- and multi-institutional studies.

## CONCLUSIONS

In summary, the MCCPR EHR-R-REDCap pipeline may help other multi-institutional registries/studies collect, aggregate, and analyze data rapidly. We provide the REDCap instrument DD, eLAB source code with instructions, key-value lookup tables as well as a simulated data set to the research community. Notably, an annotated R-markdown detailing where end users may need to adjust upfront script is also provided to assist with modifications/adaptability at other sites depending on the input format of the data source.

## FUNDING 

The Harvard Cancer Center Merkel Cell Carcinoma Patient Registry is supported by Project Data Sphere (SZS, DMM) and grants from the American Skin Association and ECOG-ACRIN (DMM). SZS was supported by the Massachusetts General Hospital Cutaneous Oncology Fellowship and the MGH ECOR Fund for Medical Discovery Clinical Research Fellowship.

## AUTHOR CONTRIBUTIONS

Sophia Z. Shalhout developed the script and was involved in the conception and design of the work and acquisition, analysis, and interpretation of the data. Farees Saqlain, Kayla Wright, and Oladayo Akinyemi were involved in the acquisition of the data. David M. Miller was involved in the conception, design, and interpretation of the work. All authors reviewed the manuscript.

## SUPPLEMENTARY MATERIAL

Supplementary material is available at *JAMIA Open* online.

## Supplementary Material

ooab118_Supplementary_Data
