# Supplementary material for: Generalizable EHR-R-REDCap pipeline for a national multi-institutional rare tumor patient registry
Source: JAMIA Open. 2022 Jan 7;5(1):ooab118. doi: 10.1093/jamiaopen/ooab118 (PMC8827011; doi:10.1093/jamiaopen/ooab118)
Supplement: ooab118_Supplementary_Data [file ooab118_Supplementary_Data.docx]

**Supplemental Materials**

**Title: Generalizable EHR-R-REDCap Pipeline for a National Multi-Institutional Rare Tumor Patient Registry**

**Authors:** Sophia Z. Shalhout^1,3^, Farees Saqlain^3^, Kayla Wright^1^, Oladayo Akinyemi^1^, and David M. Miller^1,2,3^

^1^Division of Hematology/Oncology, ^2^Department of Dermatology, Massachusetts General Hospital, MA, USA.

^3^Harvard Medical School, Boston, MA, 02114 USA

**Supplemental Materials:**

**EHR Labs Abstraction Pipeline (eLAB) Details:**

eLAB was developed in R (version 4.0.3) to re-configure structured data extracted from the EHR for REDCap-ready import into registries/repositories utilizing the provided pre-configured DD. eLAB takes as input bulk lab data pulled from the EHR. The lab data is then parsed, split, wrangled, filtered, and transformed by eLAB script on only those specific labs that are collected by our registry based on the key-value match/filtering table. eLAB remaps the labs data and units using the key-value match/filtering table and retains the labs of interest and the units of interest accepted by our registry data dictionary. Furthermore, eLAB only keeps strings/formats acceptable by our registry data dictionary. For example, script in eLAB is designed to exclude those labs with certain results such as “REFUSED LAB”/”CANCELLED” when a numeric value is pre-designated as the appropriate data type for that lab type.

The provided source code at <https://github.com/TheMillerLab/eLAB> is fully annotated to describe how each of these steps is performed by the relevant eLAB script. First, eLAB requires the bulk-pulled lab data to be loaded into the R environment and then renames the csv file as the object, “dt” in R (Figure 2). An accompanying sample “bulk EHR” dataset for user testing is also provided. After loading and renaming the data, eLAB script reconfigures columns with dates and times in the data table as ‘Y/M/D/H/M/S’ format. eLAB then splits and parses the data by separating units from values based on delimiters. Data is then remapped and filtered based on look-up tables to retain only labs and units the registry collects. These are pre-defined by the accompanying DD and eLAB reconfigures outputs/units not accepted by the registry. The data is then de-identified by using a key-value lookup table to remove patient names/medical record numbers with record identification numbers (Figure 2). Sample de-identification look-up tables are also provided for the end user to test eLAB. Final cleaning of data and data pivot from long-to-wide format transforms the data to match the strict REDCap-ready importation rules/allowances. If no errors are detected, data can be successfully imported into REDCap. Users are provided the DD for setup at their institutional REDCap site. Once imported, eLAB allows analysis of lab data with survival outcomes when linked with the full registry data elements. eLAB, for example, performs univariate analysis using the R packages survival and survminer to perform cox proportional hazard modeling on labs set to baseline. Baseline is defined as labs within a month of the date of diagnosis of MCC, and the overall survival of the patient, defined as the time from date of diagnosis and death/last follow-up visit. The provided DD also contains data fields to capture these elements manually by users. By standardizing the DD, REDCap instrument, and providing the source code, this pipeline promotes interoperability for aggregated multi-institutional collaborative studies and analysis.

**Data Dictionary (DD):**

The DD is provided for use by researchers at REDCap-participating institutions and is optimized to accommodate the same lab-type captured more than once on the same day for the same patient (<https://github.com/TheMillerLab/eLAB>). To aid manual data capture of unstructured data, the instrument was designed with conditional branching/skip-logic, where the lab tests not performed on a patient are hidden. Maximum/minimum limits were set to warn of entry errors. Once uploaded into the participating REDCap institution’s site, the DD creates the Labs electronic data capture system designed to house the data that eLAB reformats and transforms. Users may also use the Labs instrument for manual entry of lab value data. Furthermore, uploading the DD also allows users to manually capture the data fields required for outcomes measurements. These fields are located in the patient characteristics and subject status and include items such as date of death and date of diagnosis. (Supplemental Figure 1 and 2)

**eLAB and Quality Assurance (QA):**

eLAB was created and refined over many iterations until no errors were detected during manual quality control of hundreds of data values as well as with REDCaps’s import scaffold. Quality control included manual EHR/REDCap validation of over two hundred data points across all 35 lab types across all sites, as well as double abstraction of partial charts of ~100 patients and double abstraction of full charts of 30 patients. (See <https://www.themillerlab.io/post/optimizing_rwd_collection-in_instrument_qc_in_redcap/> , <https://www.themillerlab.io/post/optimizing_rwd_collection-qi-initiative-error_injection_tests/> for more details about the QA methods of the MCCPR). The eLAB code was finalized for registry use when no errors were observed during validation, double abstraction, and the import flag system.


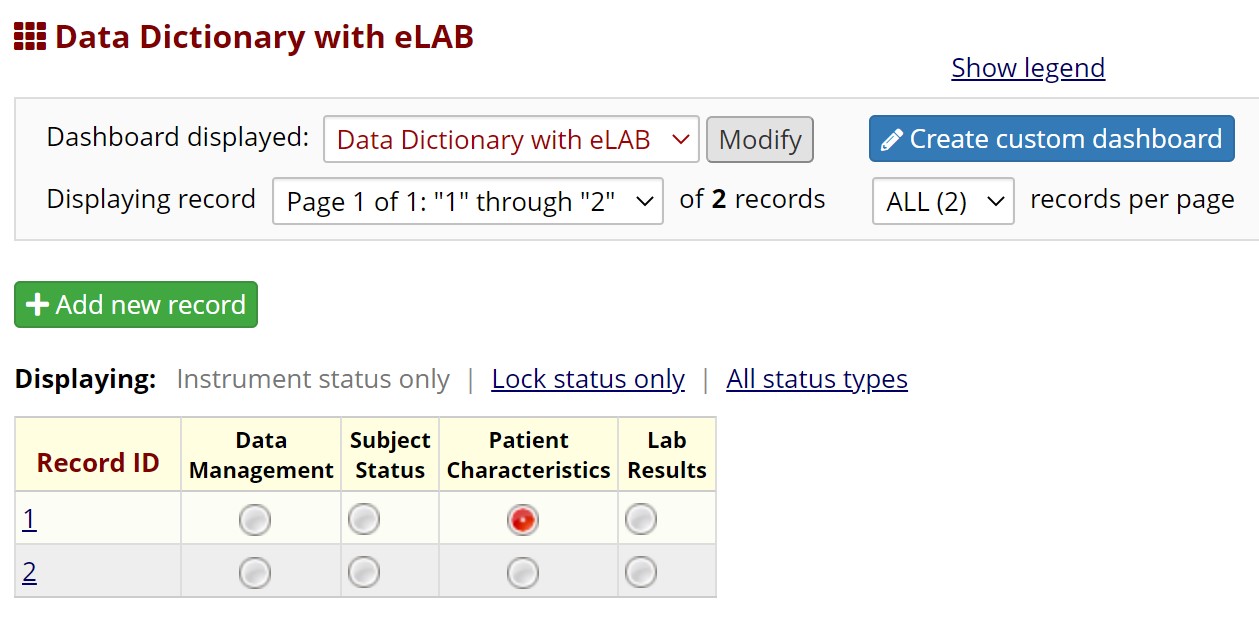


**Supplemental Figure 1: The Electronic Data Capture Interface upon uploading eLAB DD**

Users may download the eLAB data dictionary from <https://github.com/TheMillerLab/eLAB> (DataDictionary_eLAB.csv). The csv file should be uploaded into a REDCap project using the “Upload your Data Dictionary file (CSV file format only)” feature, at any REDCap participating institution. Choose the delimiter ‘comma’ for upload. Upon uploading, the user has to commit the changes. Shown here is the electronic data capture system in REDCap upon uploading eLAB DD. The ‘Lab Results’ instrument can be used to upload eLAB reformatted and transformed bulk data. Manual entry of lab data is also possible, and the user interface/user experience has been optimized for manual entry as well. Data fields required for outcomes measurements, including patient date of diagnosis and date of death are found in the subject status and patient characteristics instruments.


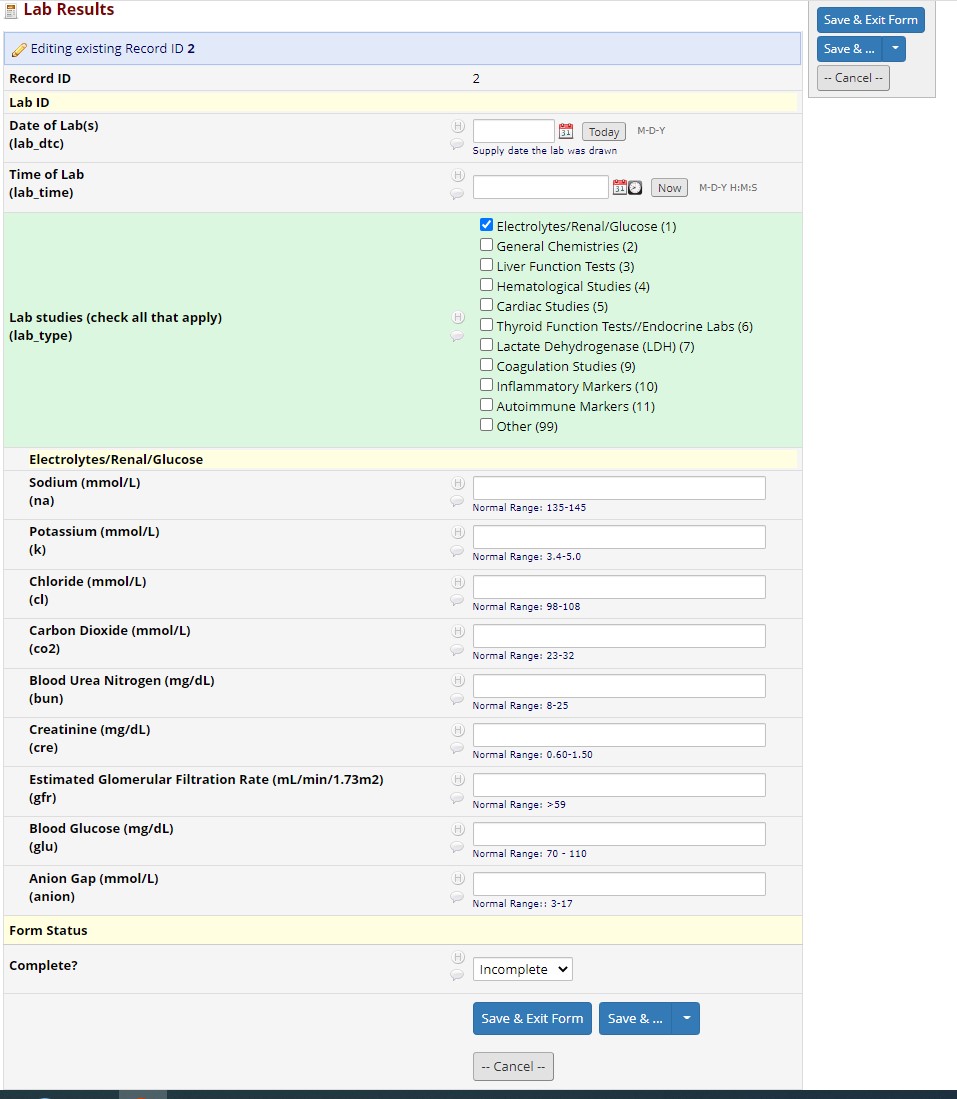


**Supplemental Figure 2: Lab Result Instrument from the eLAB Data Dictionary** Shown is the user interface of the REDCap “Lab Results” instrument after uploading the eLAB data dictionary. Bulk data pulls of laboratory values from the EHR can be remapped and transformed using eLAB and then imported and housed in the REDCap ‘Lab Results’ instrument. This instrument may also be used to capture lab data manually. To aid manual data capture of unstructured data, the instrument was designed with conditional branching/skip-logic, where the lab tests not performed on a patient are hidden. For example, in this figure, only “Electrolytes/Renal/Glucose” are checked off as the current labs of interest. Therefore, branching logic only shows those relevant labs for data entry and “general chemistries” and other unchecked lab types are hidden from view.
